# Characteristic Test Analysis of Graphene Plus Optical Microfiber Coupler Combined Device and Its Application in Fiber Lasers

**DOI:** 10.3390/s20061645

**Published:** 2020-03-16

**Authors:** Yang Yu, Hao Chen, Zhenfu Zhang, Dingbo Chen, Jianfei Wang, Zhengtong Wei, Junbo Yang, Peiguang Yan

**Affiliations:** 1 Department of Physics, College of Liberal Arts and Sciences, National University of Defense Technology, Changsha 410073, China; zhangzhenfu198206@163.com (Z.Z.); chendingbo15@nudt.edu.cn (D.C.); yangjunbo@nudt.edu.cn (J.Y.); 2 Deep Sea Technology Laboratory, College of Meteorology and Oceanology, National University of Defense Technology, Changsha 410073, China; wangjianfei09@nudt.edu.cn; 3 Shenzhen Key Lab of Laser Technology, College of Optoelectronic Engineering, Shenzhen University, Shenzhen 518060, China; hchenhao@szu.edu.cn (H.C.); yanpg@szu.edu.cn (P.Y.); 4 Department of Basic Education, Information Engineering University, Zhengzhou 450000, China; weizhengtong1987@126.com

**Keywords:** optical microfiber coupler, graphene, fiber laser, filter

## Abstract

In this study, a graphene and optical microfiber coupler (OMC) integrated device (GOMC) was proposed and fabricated. After its characteristic analysis and testing, it was applied to the development of adjustable multi-wavelength fiber lasers. By integrating the OMC with graphene, the polarization dependence of OMC was enhanced. Meanwhile, the novel GOMC was given the capabilities of filtering, coupling, beam splitting, and polarization correlation. When the GOMC was integrated as a filter and beam splitter into the ring cavity of the fiber laser, the proposed GOMC-based fiber laser could achieve single-wavelength and multi-wavelength regulated output. The laser had a 3 dB linewidth of less than 30 pm, a signal-to-noise ratio of approximately 40 dB, and an output power fluctuation of less than 1 dB. The GOMC could also be used for the development of functional devices, such as adjustable mode lockers and mode coupling selectors, which provide an excellent experimental platform for new fiber lasers and the research of multi-dimensional light-field manipulation.

## 1. Introduction

In recent years, owing to the advantages of easy integration with existing optical systems and the high sensitivity to external environmental changes, optical microfiber couplers (OMCs) have been widely used in sensing fields and other functional devices (especially by the fusing and tapering of two conventional fibers) [1,2,3,4,5,6,7,8]. Moreover, the OMC is a wavelength-dependent device that can be used as a mode-selection filter for fiber lasers [5]. Compared with the traditional filters, OMC-based fiber filters possess the advantages of easy fabrication, low cost, compact configuration, multi-transmission ports, and especially flexible structure design to realize different filter functions. In addition, due to the high heat transfer efficiency of the waist region, the optical transmission characteristics of the OMC are extremely sensitive to temperature changes [6]. Benefiting from the above-mentioned advantages, we developed an all-optical modulator and an all-optical tunable filter using OMCs [7,8]. Recently, it has been reported that mode-selective coupled OMCs can also be used in a vector fiber laser system [9]. Since the OMC has a strong function expansion, this leads to a great application value in the development of new fiber lasers. However, the transaction always has a potential disadvantage. When the OMC is integrated into the resonator of the fiber laser as a functional device, such as a filter or a beam splitter, if the package and temperature control are not performed effectively, the stability of the laser will be affected by the environmental sensitivity of the functional device. Therefore, further studies on functional devices based on OMCs are essential for different applications.

In the past ten years, two-dimensional (2D) materials, especially graphene, has attracted attention in the applications of electronics, photonics, and optoelectronics, thanks to their tunable Fermi level, saturable absorption, broad-spectrum band filling effect, ultrafast electron migrating rate, higher third-order nonlinear coefficients, and other excellent photoelectric characteristics [10,11]. In addition, 2D materials also have an ultra-high heat transfer efficiency such that they are also ideal materials for temperature control [12]. Due to the large evanescent field transmission properties of optical microfibers (OMs), its integration with 2D materials can effectively enhance the interaction between light and materials, which have been developed into fiber optic functional devices, such as polarizers, modulators, and saturated absorbers (mode lockers) [13,14,15,16]. On one hand, these functional devices are easy to integrate with existing fiber optic systems, and on the other hand, they make full use of the unique optical characteristics of two-dimensional materials to effectively implement multi-dimensional regulation of the light-field. Moreover, the cross-fusion of micro-nano photonics, photodynamic tuning, and macro-optical interconnection can also be achieved. However, in practical applications, single-port optics do not fully meet the integration requirements of various optical systems, while OMCs do meet these requirements, which can be directly tapped into using two or more fibers. In addition, compared to OMs, OMCs not only have evanescent field transmission characteristics, but also have wavelength and polarization properties. The characteristics of this composite structure light field and functional devices that include the dual-waveguide coupling and evanescent field transmission, as well as the interaction of light fields with two-dimensional materials, have not yet been reported. This work will help to further understand the interaction characteristics of the light field and two-dimensional materials, and it is expected that new devices and application prospects can be obtained.

In view of this, a graphene-OMC integrated device (GOMC) was fabricated and its optical characteristics were investigated and characterized in this paper. Then, we used the GOMC in the development of adjustable multi-wavelength fiber lasers. By integrating the OMC with monolayer graphene, the polarization dependence of the OMC was enhanced. The developed GOMC had the characteristics of filtering, coupling, splitting, and polarization correlation. It is foreseeable that based on the GOMC, all-optical tunable mode lockers, filters, and mode-coupled selectors with better temperature control effects can be developed. In addition, it can provide optional functional devices and experimental platforms for the development of new fiber lasers and multi-dimensional light-field control research.

## 2. Fabrication and Characteristic Analysis of the GOMC

### 2.1. Fabrication of the GOMC

**Figure 1:**
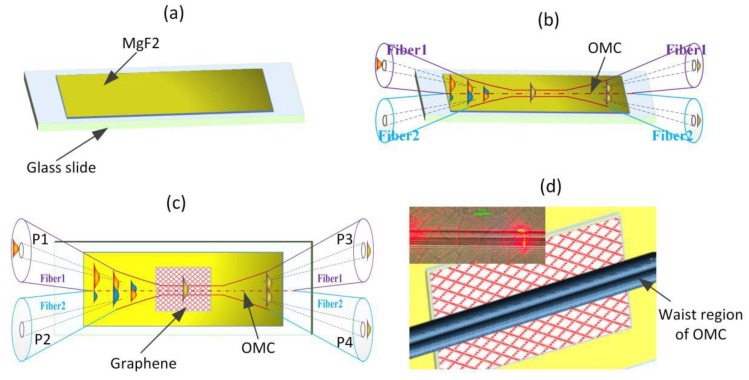
The preparation process of the GOMC: (**a**) evaporating MgF_2_ onto the glass slide, (**b**) transfer graphene to the MgF_2_ film, (**c**) the OMC was applied to a slide loaded with the graphene, and (**d**) the structure diagram and microscope photograph (insert image) of the OMC waist area and graphene bonding part.

The GOMC proposed in this paper was made of a sandwich structure, in which graphene was first placed on a glass slide, and then the OMC was fixed on the glass slide. The manufacturing process of the GOMC is shown in Figure 1. First, to prevent the loss induced by the relatively high refractive index of the glass slide, a layer of MgF_2_ with a low refractive index (1.376) and a thickness of ≈200 nm was evaporated onto it; then, a monolayer graphene sheet (after dissolving the Cu foil and removing the Polymethylmethacrylate (PMMA) film, 5 × 5 mm^2^) was mechanically transferred to the surface of the MgF_2_ [13]. The graphene film was directly synthesized using a chemical vapor deposition (CVD) method on the polycrystalline Cu substrate. Finally, the OMC was attached to the slide and the OMC waist area was aligned with the area where the graphene was located. Under the action of electrostatic forces, the waist region of the OMC was bonded to the graphene. The micrograph and structure of the junction of the OMC and graphene are shown in Figure 1d. The red scattering points at the GOMC waist region were caused by the contaminants during the sample transfer procedure (for the convenience of tracking display during the photographing process, red light was passed through the fiber). The OMC was fabricated by fusing and tapering two twisted conventional communication fibers, a method that was based on the improved flame-brushing method [6,7,8], which is comprised of a taper region and a uniform waist region. The schematic diagram of the composition OMC is shown in Figure 1c, where the P1, P2, P3, and P4 represent the four output ports of the OMC. Herein, the OMC sample for making the GOMC had a uniform waist length of 5 mm, a waist region diameter of 4 μm (i.e., the radius of the waist microfibers was 1 µm), and a lower insertion as low as 0.1 dB. This type of GOMC preparation process is simple and easy to operate, and the graphene film is sandwiched between the MgF_2_ substrate and the OMC. 

### 2.2. Characteristic Analysis and Test of the GOMC

As can be seen from the GOMC fabrication process described above, the OMC sample used was based on the improved flame-brushing method. The total coupling of the OMC was composed of the transition region coupling and the uniform waist region coupling. According to the local coupled mode theory, the output light intensity of the two output ports of the OMC (P3 port, P4 port) can be expressed as [5,6,7,8]:

(1) $$\left\{ \begin{array}{l} P_{3}=P_{0}\cos^{2}\left(\int_{0}^{l}c\left(\lambda ,n_{2},n_{3},z\right)dz\right)=P_{0}\cos^{2}\phi ,\\ P_{4}=P_{0}\sin^{2}\left(\int_{0}^{l}c\left(\lambda ,n_{2},n_{3},z\right)dz\right)=P_{0}\sin^{2}\phi , \end{array}\right.$$

where *P*_0_ is the input light power; *l* is the coupling length; *c*(*λ*, *n*_2_, *z*) is the coupling coefficient at wavelength *λ* and location *z*; and *n*_2_ and *n*_3_ are the refractive indexs (RIs) of fiber cladding (silica) and external environment, respectively. In fact, a thinner transition region and the uniform waist region of the OMC allow for its mode-coupling and spectral-transmission characteristics [5], and the GOMC proposed in this paper was made by bonding the waist region of the OMC with graphene. It can be seen from Equation 1 that the output spectrum of the two output ports (P3, P4) of the OMC should satisfy the conjugate symmetry relationship. The presence of the graphene absorbs part of the light-field energy, which is converted into the energy used to heat the optical fiber and the surrounding medium (MgF_2_, glass, and air), resulting in the changes of *n*_2_ and *n*_3_. Therefore, the absorption of optical radiation in graphene could change (via a heating effect) the attenuation and coupling characteristics of the GOMC. It is worth noting that the presence of graphene increases the heat conduction area of the OMC waist region, transferring the heat to the surrounding media, such as air and glass, and thus helping to cool the OMC waist region [6].

**Figure 2:**
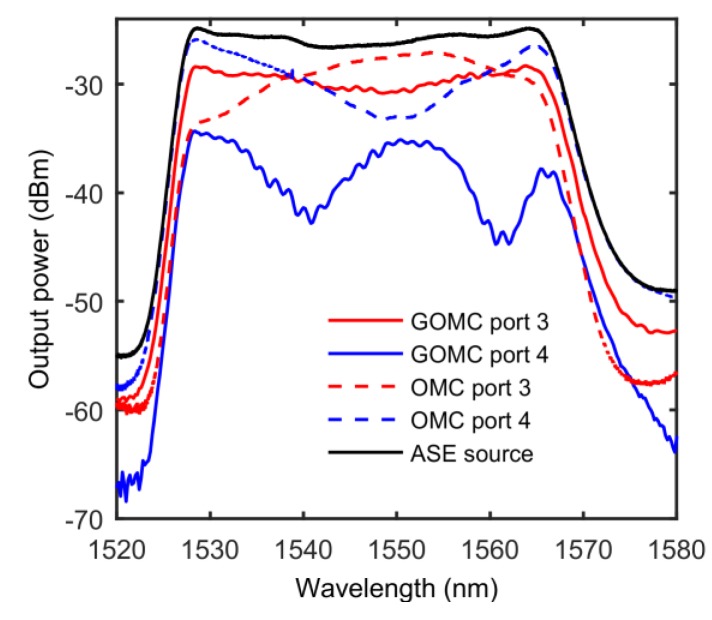
The transmission spectrum of the optical microfiber coupler (OMC) and the graphite-integrated OMC (GOMC) sample. ASE: Amplified Spontaneous Emission.

The generation mechanism of the GOMC device’s polarization properties was different from previous coupled-mode systems [17]. Its polarization correlation was not only related to the asymmetry of the OMC, but also to the light absorption of graphene, which gave the device more unique polarization characteristics. For the GOMC, the presence of graphene broke the cross-sectional symmetry of the combined device. Since the OMC had a large evanescent field similar to OM, part of the light field penetrates the graphene sheet into the surrounding environment (e.g., glass slide, MgF_2_). The presence of graphene led to different absorption losses in different modes (light fields with different polarization directions), which mad the device exhibit a relative extinction ratio. This can be seen from the transmission spectrum test of the OMC sample and the GOMC sample, as shown in Figure 2. Therefore, compared with the OMC, the GOMC had different spectral transmission characteristics, which led to the output spectrum of the two output ports no longer satisfying the conjugate symmetry relationship. Therefore, the GOMC became a natural broad-spectrum polarization-dependent device.

**Figure 3:**
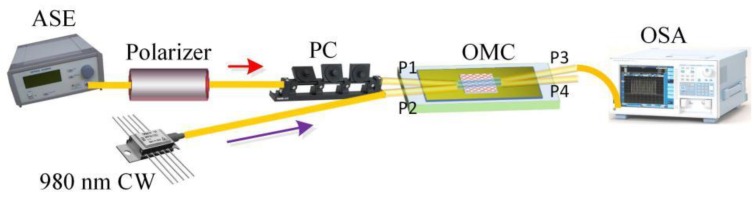
The schematic diagram of the GOMC transmission characteristic test system. CW: 980-nm Continuous Wave, OSA: Optical Spectrum Analyzer, PC: Polarization Controller.

The polarization characteristics of fiber optic devices have a huge impact on their performance and system integration applications. In this work, the polarization and spectral transmission characteristics of the proposed GOMC composite device were experimentally studied, where the schematic diagram of the measurement system is shown in Figure 3. Because the amplified spontaneous emission (ASE) light source was non-polarized, we connected the source to the GOMC via an in-line fiber polarizer and a polarization controller (PC). Then, by adjusting the PC, different eigenmodes (polarization states) could be injected into the device to allow for the testing and analysis of the polarization transmission characteristics of the GOMC. The spectral transmission characteristics of the GOMC were analyzed using an optical spectrum analyzer (OSA; 600–1700 nm; Q8384, ADVANTEST, Japan; the resolution was 0.01 nm). In this study, the GOMC sample used in the experimental test had a splitting ratio of 3:1, corresponding to an output intensity ratio of 3 for the P3 and P4 ports. The device insertion loss of our GOMC was about 3 dB. 

**Figure 4:**
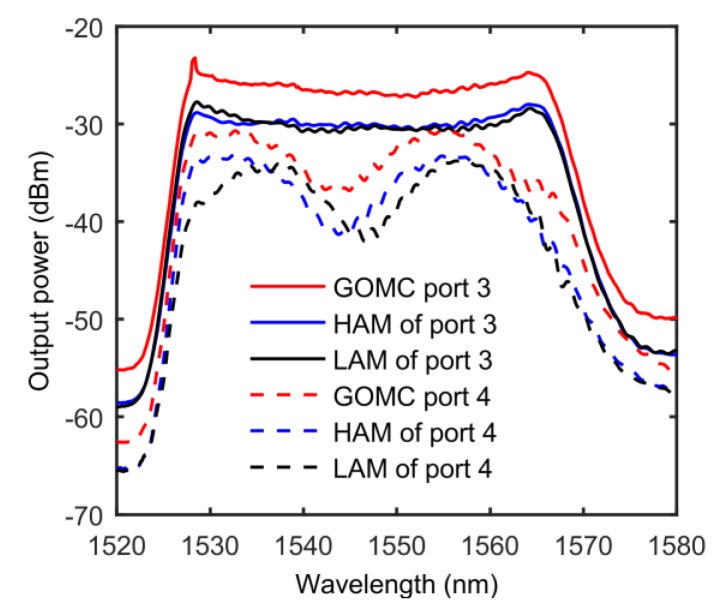
The spectral transmission characteristics of the GOMC. HAM: High-Absorption-Loss Mode, LAM: Low-Absorption-Loss Mode.

During the experiment, we connected the ASE directly to the GOMC to test the intrinsic spectral transmission characteristics of the device. The test results of the GOMC spectral transmission characteristics are shown in Figure 4, in which the red solid line and the dotted line are the characteristic spectra from the P3 and P4 port outputs, respectively. Comparing Figure 2 and Figure 4, it can be seen that the characteristic spectra of the GOMC and the OMC were completely different, and neither met the conjugate symmetry nor regular periodic oscillation conditions. This shows that under the action of graphene and the glass slide, the mode transmission characteristics of the OMC waist region could not be considered two-mode interference. Moreover, during the OMC sample preparation, the twisting and tapering may have caused tolerances in the spacing of the two coupled microfibers, resulting in different coupling coefficients for the through port and the coupled port. However, from previous experience, the geometric asymmetry of the GOMC and the natural absorption of graphene were the main factors leading to the non-conjugation of the characteristic spectrum of the device.

In addition, the ASE power input at the experiment was fixed at 10 mW. By adjusting the PC to inject two different polarization states (herein referred to as the low-absorption-loss mode (LAM) and the high-absorption-loss mode (HAM)) into the GOMC, the characteristic spectrum of the device was significantly shifted (i.e., red-shifted, the spectrum shift was due to the change of polarization state). When the GOMC was injected into the LAM and the HAM, the output intensity difference of the device was about 5 dB at 1550 nm, which was attributable to the broken symmetry of the GOMC geometry and the absorption of the graphene. It can be seen that the GOMC was very sensitive to the polarization and became a typical polarization-dependent device. In view of this, if the GOMC were to be combined with a PC, the tunable filtering function could be conveniently developed.

**Figure 5:**
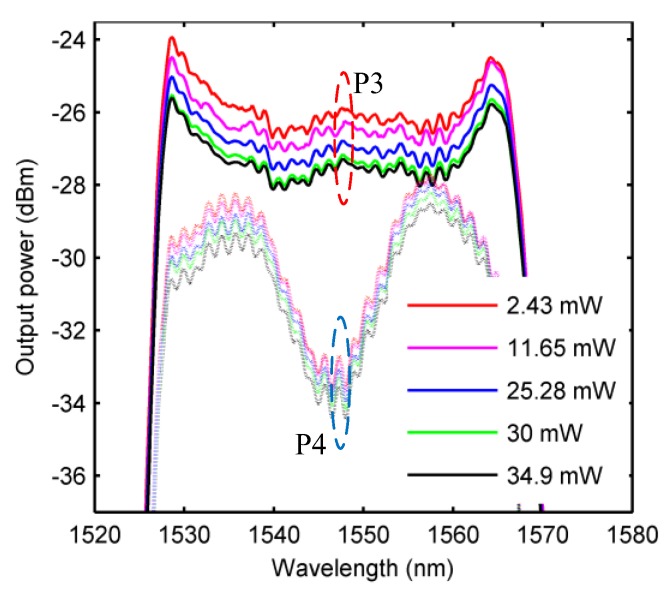
The all-optical modulation test results of the GOMC.

In order to further understand the temperature control characteristics of the device, the all-optical modulation method was employed to test and analyze the device. The internal light absorption heating effect was using to regulate the spectral transmission characteristics of the GOMC [6,7,8]. First, using the experimental system shown in Figure 3, different intensity "DC" 980-nm continuous pump light was injected into the device to test the "static" all-optical modulation characteristics of the polarization-dependent GOMC platform. During the experiment, the power of the ASE probe light injection device (10 mW) and the polarization control state of the PC were kept unchanged. The test results are shown in Figure 5. As the pump power was increased, the output spectra of the two output ports of the GOMC showed an overall attenuation, i.e., the device insertion loss rose while the modulated optical power was increased. The test results showed that when the pump power of 980 nm was increased from 2.43 mW to 34.9 mW, the overall attenuation of the output spectra of the P3 and P4 port were about 1.5 dB and 0.9 dB, respectively. However, the characteristic spectrum of the GOMC did not shift. This was different from the tunable filter introduced in Yang et al. [8]. Under the action of internal light absorption and heating, the GOMC could not achieve tunable filtering and its filtering characteristics were stable.

The light absorption of the GOMC was mainly from two aspects: waveguide intrinsic absorption of the fiber and graphene, and the PMMA of the graphene residue and adhesion of air pollutants during the sample preparation (the part of the energy of these absorbed light was converted into heat). Under the action of the thermo-optic effect, the OMC evanescent field ratio increased, which in turn caused the GOMC insertion loss to increase. However, the characteristic spectrum of the GOMC did not shift significantly during the modulation process. Therefore, it can be inferred that the extrinsic absorption of impurities was an important factor that caused the device loss to increase with the increase of the modulated optical power. In view of this, to improve the device stability and temperature control characteristics, various impurity contaminations introduced by the device fabrication process should be removed as much as possible. It should be noted that, unlike in Jin et al. [13], the detection optical power used in the experiment was relatively large (the intensity of the ASE injection device was equivalent to the modulated optical power); therefore, the intensity modulation phenomenon due to the saturated absorption characteristics of the graphene was not observed. According to the Pauli exclusion principle, it is necessary to use a short-wave, high-power pump light to control the long-wave weak probe light [13]. In fact, this experiment paid more attention to the effects of light absorption and heat on the transmission characteristics of the device to illustrate the thermal regulation characteristics of the GOMC. 

The foregoing experiments showed that the characteristic spectrum of the GOMC did not shift significantly during the static pump light modulation. This came down to two factors. First, the two OMs of the OMC waist region had a large surface-to-volume ratio (the OMC’s waist area can be regarded as two optical microfibers connected in parallel, as shown in Figure 1b), and its full adhesion to graphene further expanded the heat conduction surface [6]. Second, the excellent heat transfer characteristics of graphene could effectively release the heat generated by the light absorption conversion, thereby ensuring that the GOMC conformed to the waveguide boundary conditions. To further verify this inference, we also used the all-optical modulation method of Yang et al. [7], which used different frequencies of "dynamic" sinusoidal intensity signals to control the pump light injection into the GOMC to further test the light absorption and heat modulation characteristics of the device. The experimental results showed that under the "dynamic" modulation of the pump light, the intensity modulation signal was not detected at the two output ports of the GOMC. This again verified the influence of graphene on the temperature characteristics of the device.

## 3. Fiber Laser Experiment and Discussion

**Figure 6:**
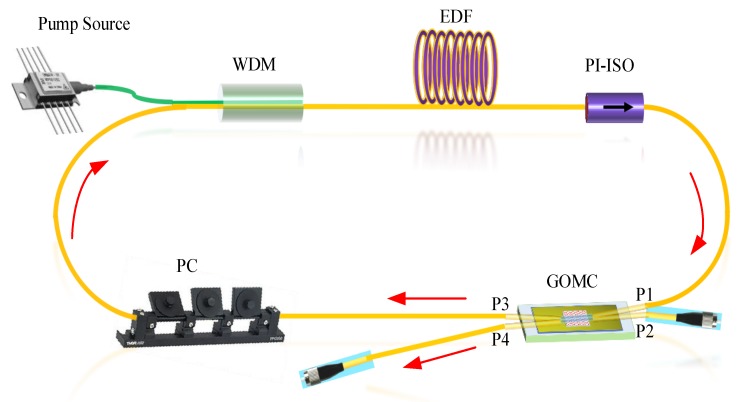
Experimental setup of the proposed laser based on the GOMC. EDF: Erbium-Doped Fiber, PI-ISO: Polarization Independent Isolator, WDM: Wavelength Division Multiplexer.

The above analysis and test of the GOMC showed that the combined device had better temperature control characteristics and was very sensitive to the polarization, which could be used as a stable filter combined with the PC to realize tunable filtering. In view of this, the GOMC sample was used in the development of tunable multi-wavelength fiber lasers to further verify and develop the functions. The ring cavity fiber laser experimental test system is shown in Figure 6. The set-up used a 980-nm laser diode (LD) as the pump source, which passed through the 980/1550-nm wavelength division multiplexer (WDM) 980 nm port and common port, which was then connected to a 5-m erbium-doped fiber (EDF). Then, the EDF was connected to the P1 port of the GOMC via a 1550-nm polarization independent isolator (PI-ISO) that had a pump light absorption efficiency of approximately 13 dB/m. The isolator ensured that the laser was amplified, the output was clockwise within the laser cavity, and the laser stability was improved. The GOMC’s P3 port was connected to the 1550-nm port of the 980/1550-nm WDM via a PC to form the complete fiber laser cavity loop. It can be seen from the above analysis and experiment that the adjustment of the PC could realize the regulation of the filtering characteristics of the GOMC, thereby realizing the multi-wavelength output of the fiber laser. The ring fiber laser had a total cavity length of about 7 m. The GOMC’s P4 port (25% port) was used as the ring fiber laser output port, which could be connected to the OSA to analyze the characteristics of the laser output spectrum. The P2 port of the GOMC could be used to monitor the spontaneous emission and mode competition characteristics within the laser. During the experiment, the GOMC was packaged in a plastic box to minimize the impact of the environment on the laser system’s stability.

**Figure 7:**
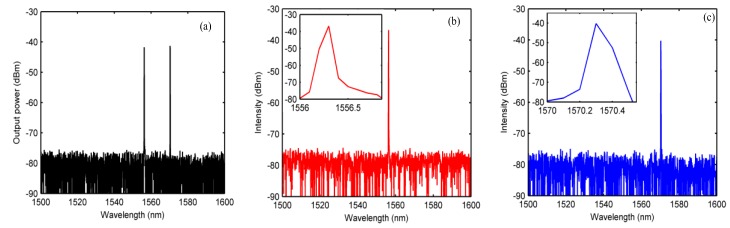
Test spectra of lasers in single-wavelength and dual-wavelength output states: (**a**) 1556-nm and 1570-nm output, (**b**) 1556-nm output, and (**c**) 1570-nm output.

By adjusting the polarization state of the PC in the laser cavity, the laser could achieve single-wavelength, dual-wavelength, or even triple-wavelength outputs. In fact, more wavelengths of laser output state can be achieved through OMC structural optimization. For example, using a long or relatively thin waist sample, a dense frequency-comb-filtering function can be obtained to meet the needs of multi-wavelength laser development [8]. Figure 7 shows the output spectrum of the laser at single-wavelength and dual-wavelength outputs when the pump power was 200 mW. As can be seen in Figure 7, the signal-to-noise ratio (SNR) was about 40 ± 2 dB in the case of a single-wavelength output, and the illustration shows that the 3 dB line-width of the output laser was less than 30 pm. The test accuracy of the 3 dB line-width here was limited by the detection accuracy of the OSA.

**Figure 8:**
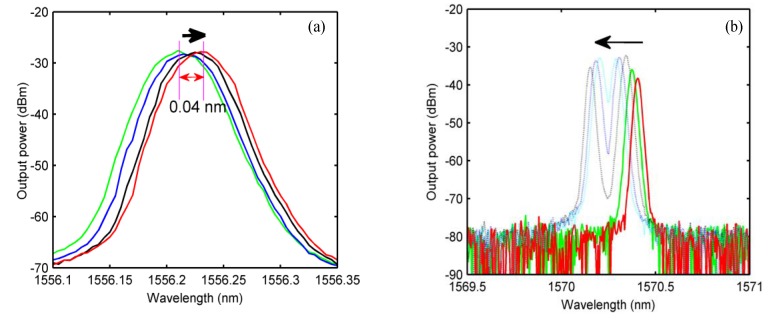
The laser output spectral test results during the fine-tuning of the PC: (**a**) rotation of the PC’s polarization control sheet clockwise and (**b**) rotation of the control piece of the PC counterclockwise.

In order to further understand the regulation of the polarization state of the PC using the laser wavelength output characteristics, we observed the laser output state after slightly selecting the PC’s polarization control chip under the single-wavelength output state of the laser. The test results are shown in Figure 8. When the laser was operating in the short-wave (1556 nm) output state, as the PC’s polarization control chip was rotated slightly clockwise, the output laser’s center wavelength appeared red-shifted (as shown in Figure 8a). When the laser was operating in the long-wave (1570.3 nm) output state, as the PC’s polarization control chip was rotated counterclockwise, the output laser’s center wavelength appeared blue shift, and then gradually split into double peaks until the dual-wavelength output state was reached (Figure 8b).

Comparing the polarization correlation test results of the aforementioned GOMC, it was not difficult to find that the polarization state change of the PC determined the overall filtering characteristics of the combined device with the GOMC, and thus the laser could be operated in different wavelength output modes. The polarization sensitivity and filtering function of the GOMC were also verified. In addition, the number of wavelengths output by this type of laser was mainly determined by GOMC filtering and insertion loss characteristics. By adjusting the length of the OMC waist region and the length of the region bonded to graphene, the GOMC samples with different filtering and insertion loss characteristics could be obtained. Therefore, the GOMC performed as a flexible device platform for the development of multi-wavelength fiber lasers.

**Figure 9:**
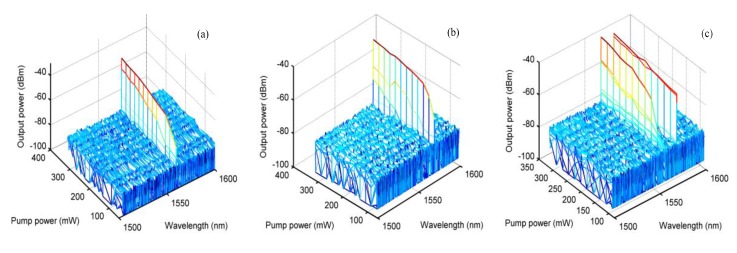
The output spectra of the laser under different 980-nm pump optical powers: (**a**) 1556-nm output, (**b**) 1570-nm output, and (**c**) 1556-nm and 1570-nm output.

During the experiment, the output spectral power stability of the laser was also tested. The test results are shown in Figure 9. In the single-wavelength and dual-wavelength modes of operation, the laser output power increased with the pump power, and the output spectrum was stable. At different pump optical powers, the laser’s working center wavelength was stable without offset. The designed GOMC-based ring fiber laser could achieve stable, intensity-tunable, multi-wavelength-tunable laser output. This also indicated that the GOMC filtering characteristics were stable. The GOMC could accommodate the dual functions of the filter and the coupler in the laser cavity, which further simplified the laser system composition. Compared to other fiber laser systems, GOMC-based fiber lasers are simple, compact, low-cost, and easy to fabricate.

**Figure 10:**
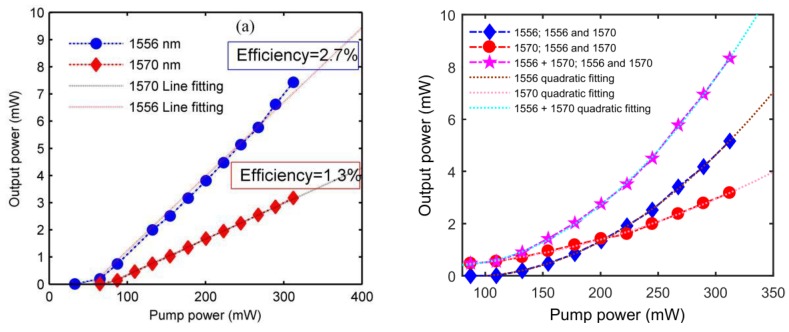
The output efficiency test results of the laser: (**a**) single wavelength output and (**b**) dual-wavelength output.

Figure 10 shows the output efficiency of the constructed fiber laser. When the laser was using a single-wavelength output, the laser output power increased linearly with the pump light. When the laser was operating at a short wavelength (1556 nm), the laser threshold was approximately 65 mW and the output efficiency was approximately 2.7%. When the laser was operating at a long wavelength (1570 nm), the laser threshold was about 87 mW and the output efficiency was about 1.3% (as shown in Figure 10a). The threshold of the laser’s dual-wavelength output was about 110 mW and the output power increased quadratically with the pump power. It can be seen that the short-wave output efficiency improved faster (as shown in Figure 10b). This indicated that the dual-wavelength output consumed a higher amount of pump light, but once the output was excited, the output efficiency increased rapidly with the pump power and was eventually as efficient as the single-wavelength output state. In addition, it can be seen from the test results that the laser cavity loss was still large. To meet practical applications, it is necessary to improve the output efficiency. The cavity loss of this laser was mainly determined by the insertion loss of the GOMC device (about 3 dB). In the sample preparation process, if the magazine residue on the graphene can be effectively removed and air pollution is avoided, a high-quality GOMC sample can be obtained. By optimizing the OMC parameter structure, the laser loss characteristics can be effectively improved.

**Figure 11:**
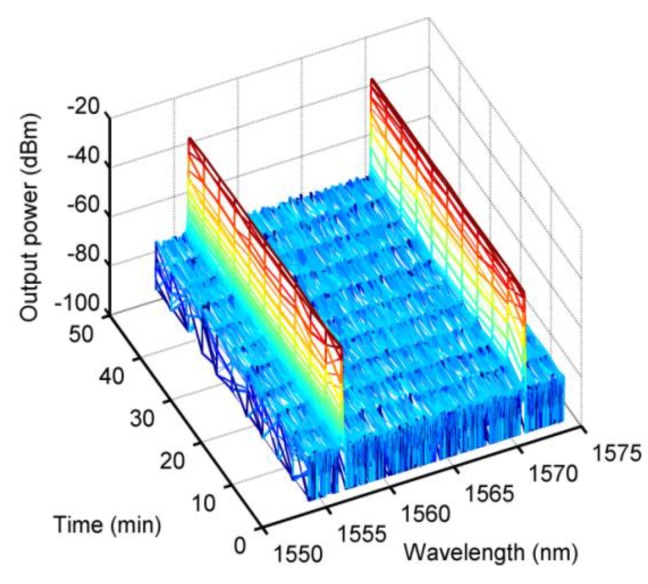
The stability test results of the laser.

We also tested the stability of the laser’s dual-wavelength output state. During the experiment, the laboratory temperature (25 °C) and humidity (15%) were kept constant. When the pump power was 200 mW, the output state of the laser was monitored for 50 minutes. The test result is shown in Figure 11. The output power fluctuation of the 1556-nm center wave was less than 1 dBm, the output power fluctuation of the 1570-nm center wave was less than 0.8 dBm, and the two center wavelengths had no offset fluctuation. This power fluctuation was mainly due to the thermal noise of the laser system. 

The laser stability test showed that the proposed laser was better than the OMC-based fiber laser introduced in Harith [5]. This indicates that the presence of graphene can improve the thermal stability of the laser system to some extent. It also shows that the GOMC composite device has better thermal regulation characteristics and can be used as a key filter function device for laser development. In addition, this stable multi-wavelength laser can be widely used in communication, sensing, microwave photon radar, and other systems; therefore, it has great commercial value. It should be noted that when the GOMC was used as a light control function device, it needed to be packaged and protected. In an open environment, graphene itself will accumulate gases, impurities, and so on. Therefore, the GOMC can also be used as a sensing platform for gas and biosensor research.

## 4. Conclusions

We proposed and manufactured a GOMC combination device. Based on the characteristics analysis and testing of the device, it was applied to the development of multi-wavelength lasers. This device took full advantage of the high thermal conductivity of graphene and the advantages of OMC filtering. It had multi-port, evanescent field transmission; obvious polarization dependence; and excellent temperature adjustment characteristics. The experimental results demonstrated that the GOMC-based fiber laser could achieve single-wavelength and multi-wavelength regulated output. The 3-dB linewidth of the laser was less than 30 pm, the SNR was about 40 dB, and the output power fluctuation was less than 1 dB. The proven GOMC-based fiber laser featured the advantages of a compact structure, easy fabrication, and stable performance. In addition, the GOMC could perform as an alternative new experimental platform for light-field control, fiber laser development, and sensing applications.
